# Quantification of the relationship between two cardiac magnetic resonance techniques: fast gradient echo and steady-state free precession for determination of left atrial volumes

**DOI:** 10.1186/1532-429X-17-S1-P19

**Published:** 2015-02-03

**Authors:** Filip Zemrak, Pierre-Jean Pintard, Elzbieta H Chamera, Saidi A Mohiddin, Saman Nazarian, Joao A Lima, Steffen E Petersen, David Bluemke

**Affiliations:** 1 Cardiology Division, Department of Medicine, Johns Hopkins Hospital, Baltimore, MD, USA; 2 Radiology and Imaging Sciences, Clinical Center, National Institutes of Health, Bethesda, MD, USA; 3 Centre for Advanced Cardiovascular Imaging, Queen Mary University of London, The London Chest Hospital, London, UK

## Background

Steady-state free precession (SSFP) results in larger volumes and lower ejection fraction for the left ventricle compared to fast gradient echo (fGRE) imaging. Similar comparisons for the left atrium (LA) are not available and may be relevant for 3T imaging and historical comparison to prior imaging datasets. As opposed to LV imaging, volumes in the LA are more commonly derived using biplane area measurements.

## Methods

Cardiac cine CMR examinations were acquired in 198 randomly selected individuals who underwent both SSFP and fGRE cardiac pulse sequences in The Multi-Ethnic Study of Atherosclerosis (MESA) population based cohort. LA volumes were measured using dedicated software (CMR42, Circle CVI, Calgary) by bi-planar technique in end-systolic left ventricular phase, just before the mitral valve opening. Paired Student t-tests were performed to evaluate the difference between the sequences. Linear regression models provided correlation estimates as well as slope and intercept estimates with fGRE predicting SSFP measures. A two-way mixed model was used to estimate the intraclass correlation coefficient (ICC) between the techniques. The limits of agreement between the fGRE and SSFP measured LA volumes were compared using the Bland-Altman analysis.

## Results

The mean LA volume measured by SSFP (65.1±21.2 ml) was 3 % larger than by fGRE (63.2±21.9 ml), p < 0.001. The relationship between SSFP and fGRE measures was linear and highly correlated (r=0.94, p < 0.001). There was an excellent agreement between two methods with ICC of 0.933 (0.908 to 0.95), confirmed also on the Bland-Altman analysis. We determined linear regression models to estimate the SSFP values based on the fGRE measures. Slope for LA volume was 0.91 and the intercept was 7.73 (SSFP LA volume = 0.91 x fGRE volume + 7.73) (Figure 2).

**Figure 1:**
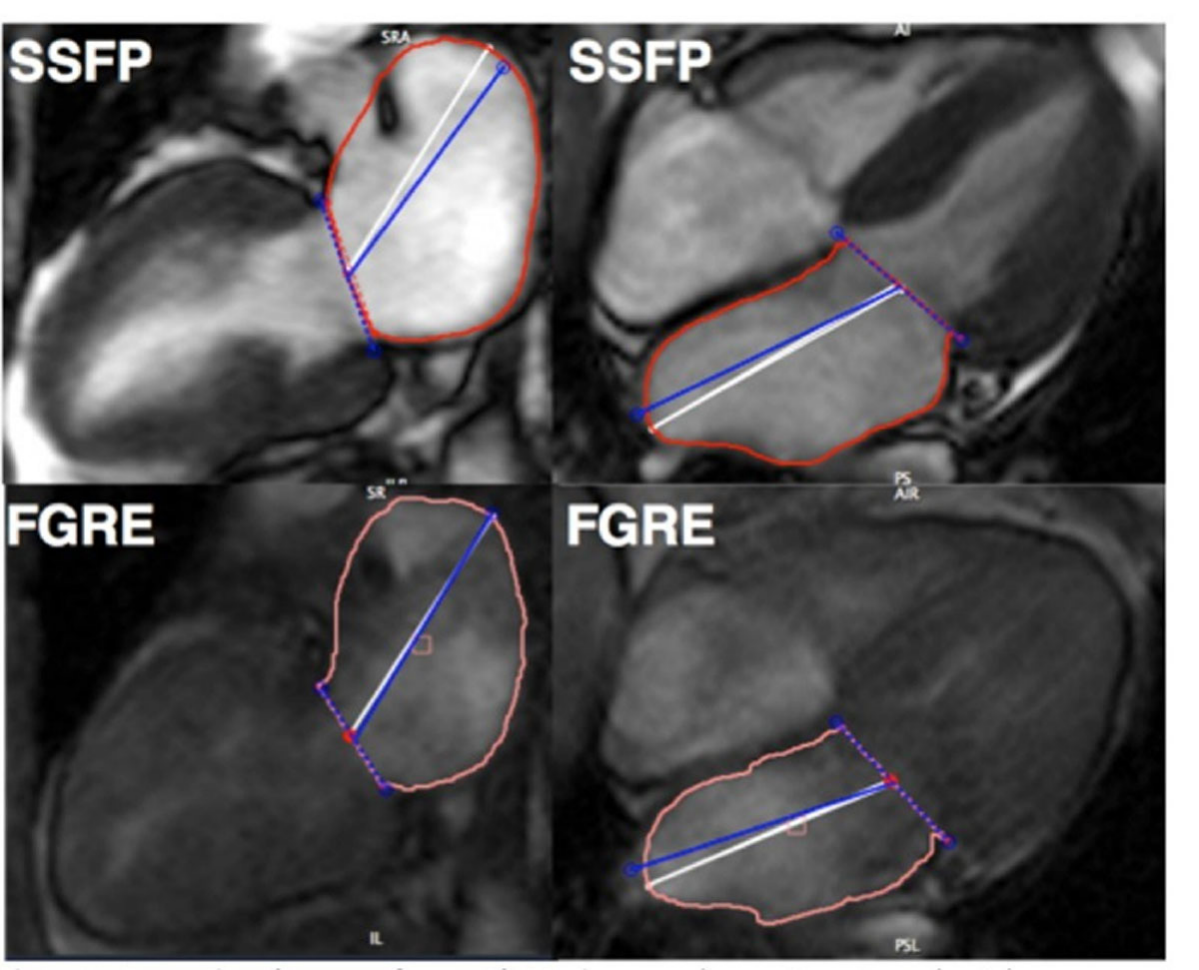
**Comparison between fGRE and SSFP images.** The two images on the right represent two-chambers (vertical) image while the images on the left provide a view of the four chambers (horizontal cine). The higher definition of SSFP images enables a more accurate calculation of the LA size.

**Figure 2:**
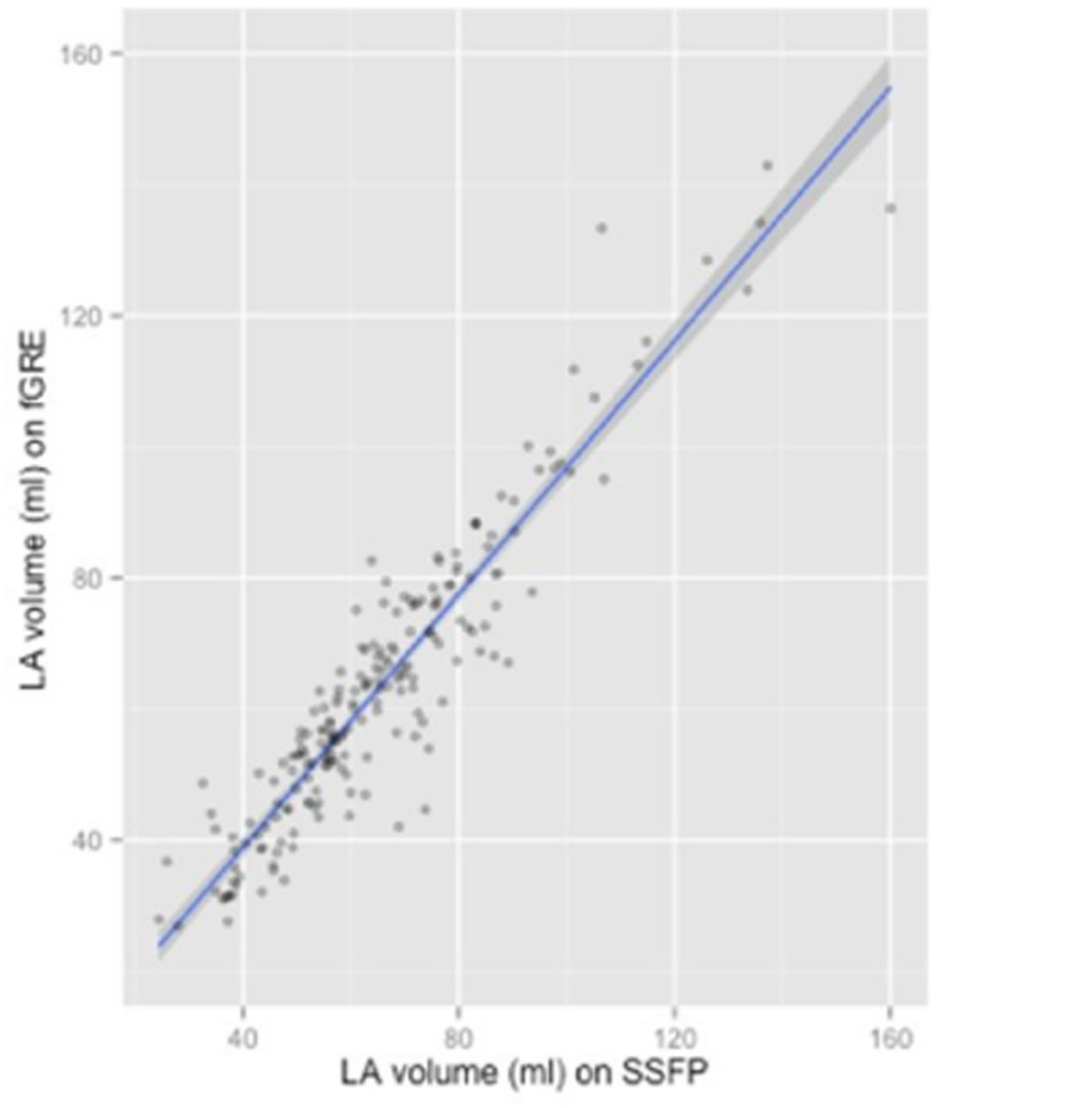
**A. Relationship between CMR fGRE and DDFP sequences for LA volume measurement.** fGRE –fast gradient recalled echo, SSFP – steady state free precession.

## Conclusions

SSFP LA volumes are approximately 3% larger than those evaluated by fGRE method using a biplanar method of measurement.

## Funding

National Institute for Health Research Cardiovascular Biomedical Research Unit at Barts (FZ, SEP) and National Institute of Health (MESA).

